# Genetic Depletion of Amylin/Calcitonin Receptors Improves Memory and Learning in Transgenic Alzheimer’s Disease Mouse Models

**DOI:** 10.1007/s12035-021-02490-y

**Published:** 2021-07-27

**Authors:** Aarti Patel, Ryoichi Kimura, Wen Fu, Rania Soudy, David MacTavish, David Westaway, Jing Yang, Rachel A. Davey, Jeffrey D. Zajac, Jack H. Jhamandas

**Affiliations:** 1 Department of Medicine (Neurology), Neuroscience and Mental Health Institute, University of Alberta, Edmonton, AB T6G 2S2 Canada; 2 Center for Liberal Arts and Sciences, Sanyo-Onoda City University, Yamaguchi , 756-0884 Japan; 3 Faculty of Pharmacy, Cairo University, Giza, Egypt; 4 Department of Biochemistry, University of Alberta, Edmonton, AB T6G 2H7 Canada; 5 Centre for Prions and Protein Folding Diseases, University of Alberta, Edmonton, AB T6G 2M8 Canada; 6 Department of Medicine, University of Melbourne, Austin HealthHeidelberg, VIC 3074 Australia

**Keywords:** Amylin, Amylin receptor, Calcitonin receptor, Alzheimer’s disease, Amyloid-β protein, Long-term potentiation, Hippocampus, Spatial memory

## Abstract

Based upon its interactions with amyloid β peptide (Aβ), the amylin receptor, a class B G protein-coupled receptor (GPCR), is a potential modulator of Alzheimer’s disease (AD) pathogenesis. However, past pharmacological approaches have failed to resolve whether activation or blockade of this receptor would have greater therapeutic benefit. To address this issue, we generated compound mice expressing a human amyloid precursor protein gene with familial AD mutations in combination with deficiency of amylin receptors produced by hemizygosity for the critical calcitonin receptor subunit of this heterodimeric GPCR. These compound transgenic AD mice demonstrated attenuated responses to human amylin- and Aβ-induced depression of hippocampal long-term potentiation (LTP) in keeping with the genetic depletion of amylin receptors. Both the LTP responses and spatial memory (as measured with Morris water maze) in these mice were improved compared to AD mouse controls and, importantly, a reduction in both the amyloid plaque burden and markers of neuroinflammation was observed. Our data support the notion of further development of antagonists of the amylin receptor as AD-modifying therapies.

**Supplementary Information:**

The online version contains supplementary material available at 10.1007/s12035-021-02490-y.

## Introduction

A defining pathological feature of Alzheimer’s disease (AD) is the presence of soluble oligomers of amyloid beta (Aβ) that aggregate into extracellular fibrillary deposits known as amyloid β-plaques [1]. Although the role of the brain Aβ in AD is not completely resolved, available evidence indicates that it may initiate and/or contribute to the process of neurodegeneration [2, 3]. Neuronal dysfunction, synaptic disruption, neuroinflammation, and vasculopathy are key perturbations of AD pathology and each is linked to the presence of Aβ in the brain [1, 3]. Aβ protein has been shown to interact with a number of G protein-coupled receptors (GPCRs) to modulate major cognitive and pathological features of AD and has thus become attractive therapeutic targets for the development of disease-modifying treatments for AD.

We have shown that human amylin, a 37-amino acid peptide identified in pancreas of diabetics, causes neurotoxicity in a manner very similar to Aβ, and that the effects of the human amylin and Aβ on neurons appear to be expressed via the same receptor, the amylin receptor, a class B G protein-coupled receptor [4]. Pharmacological studies using the amylin receptor antagonist, AC253, have shown beneficial effects on synaptic function, spatial memory and learning, and neuroinflammation in transgenic mouse models of AD [5–7]. However, other studies have reported that the systemic administration of human amylin or pramlintide, a synthetic non-amyloidogenic analog of amylin, is capable of improving deficits in spatial memory [8, 9]. Thus, to address these contradictory findings arising from the pharmacological evidence, we generated a line of transgenic (Tg) AD mice expressing a genetically induced deficit in amylin/calcitonin receptors, but carrying the full amyloid protein precursor (APP) gene and resultant overexpression of Aβ. If the amylin receptor is indeed a mediator of deleterious effects of Aβ, one would expect a significant reduction and/or delay in the development of age-dependent pathology and memory-related deficits in the AD mice bearing a reduced complement of amylin receptors compared to AD mice.

Dimerization of the calcitonin receptor (CTR) with one of the three receptor activity-modifying proteins (RAMP1, RAMP2, or RAMP3) yields subtypes of amylin receptors (AMY1-3) that bind amylin with varying affinities [10, 11]. As such, CTR is a critical component for the functionality of the amylin receptor, i.e., without CTR, there is no functional amylin receptor even if RAMPs are present. We have previously shown that in cell cultures of human fetal neurons, siRNA downregulation of CTR blunts Aβ-induced cell death [4]. Thus, to extend these in vitro findings, we sought to genetically deplete CTR receptors in the transgenic AD mice. Since homozygosity for CTR null alleles is embryonic lethal, we sought to assess the impact of hemizygosity for the CTR locus. We thus obtained stocks of CTR mice with one inactivated CTR allele (HetCTR) [12]. These HetCTR mice, producing 50% of the amylin receptor complement of wild-type (WT) mice, were crossed with two strains of transgenic AD mice. We used TgCRND8 and 5xFAD lines of mice, both of which express APP695 and develop Aβ-related neuropathology consisting of large numbers of diffuse plaque amyloid deposits in tandem with a progressive deterioration of spatial memory [13, 14]. Crossing such hemizygous null CTR (HetCTR) mice with TgCRND8 or 5xFAD mice produces HetCTR + TgCRND8 or 5xFAD compound Tg mouse offspring, which have 50% depletion of amylin receptors but will continue to overexpress Aβ deriving from the separate APP695 transgene array. Thus, the compound mice could be used to test whether or not a constitutive genetic depletion of amylin receptors alters the AD-related pathology and memory-related deficits that follow from expression of FAD alleles of human APP695. More generally, this approach allows a critical assessment of some contradictory pharmacological findings and the potential benefits of modulating the AMY/CTR axis in clinical dementia settings.

## Materials and Methods

All experiments were conducted in compliance with the guidelines set by the Canadian Council for Animal Care and with the approval of the Human Research Ethics Board and Animal Care Use Committee (Biomedical Sciences) at the University of Alberta (Protocol AUP00000268).

### Chemical and Reagents

All commercially available chemicals were of analytical grade and used without further purification. Oligomeric Aβ_1-42_ was prepared according to published protocol [15]. Briefly, Aβ_1-42_ (rPeptide) was dissolved to 1 mM in 100% hexafluoroisopropanol, hexafluoroisopropanol was removed under vacuum, and the peptide was stored at − 20 °C. For oligomeric conditions, the peptide was first re-suspended in DMSO to 5 mM, then water added to bring it to a final concentration of 1 mM, and the peptide incubated at 4 °C for 24 h. The hAmylin (AnaSpec Inc.) was dissolved to 1 mM in water and kept at − 80 °C. Aliquots of Aβ and hAmylin were further diluted to final application concentration with cell culture medium.

### Animal Models

Mice were maintained in standard laboratory housing conditions (20 ± 1 °C; 70% ± 10% humidity; 12:12 h light/dark cycle). Access to standard rodent chow and water was available ad libitum.

For behavior and histological studies, we used non-Tg (C57BL/6xC3H background) and TgCRND8 mice (human APP695 transgene array incorporating Swedish K670M/N671L and Indiana V717F mutations superimposed upon a C57BL6xC3H genetic background), which have been described previously to exhibit Aβ plaques and cognitive deficits from 3 months of age onwards [13]; these mice originating from the University of Toronto are being deposited at the Jackson Lab, Bar Harbor, ME (JAX # 020,661). 5xFAD amyloid precursor protein (APP)/presenilin 1 (PS1) double transgenic mice with five familial AD mutations [14] mouse breeding stocks were obtained from the Jackson Laboratory (JAX #006,554) and correspond to a C57BL6xSJL hybrid background. Additionally, heterozygous CTR (HetCTR) age-matched mice (C57BL/6 J background) with a 50% depletion of CTR expression were obtained using breeding pairs provided from Drs. RA Davey and JD Zajac (University of Melbourne, Australia), these being obtained as C57/BL6 congenic stock [15]. Crosses of HetCTR and heterozygous Tg-positive mice (5xFAD or TgCRND8) were set to obtain compound heterozygotes (i.e., hemizygous for a functional CTR locus and heterozygous for carrying the APP-expressing transgene array). Both male and female mice were used in our study.

Early and late stage male and female AD mice with and without CTR depletion and their WT littermate controls were evaluated at approximately 4 and 8–12 months of age, respectively, and described henceforth as 4 and 8 months of age. In both cases, the animals were stratified by age to maintain equivalent age distributions between WT and transgenic experimental groups.

### Genotyping Mouse Lines

*TgCRND8* mice were genotyped using PCR primers in the human APP coding region and the hamster PrP 3’-untranslated region, 5’.TGTCCAAGATGCAGCAGAACGGCTACGAAAA.3’, and 5’.AGAAATGAAGAAACGCCAAGCGCCGTGACT.3’. PCR with Taq polymerase (Invitrogen) was performed with a 94 °C melt step (180 s) followed by 35 cycles using a 94 °C melt temperature (20 s), hybridization at 68 °C (20 s), and extension at 72 °C (90 s).

*5xFAD* mice were genotyped with APP and presenilin 1 primer sets.

APP 5’.AGGACTGACCACTCGACCA.3’ transgene forward and 5’.CGGGGGTCTAGTTCTGCA T.3’ transgene reverse, with 5’.CTAGGCCACAGAATT GAAAGA TCT.3’ internal positive control forward and 5’.GTAGGTGGAAATTCTAGCATCATC.3’ internal positive control reverse primer. The APP amplicon is 377 bp, and the control amplicon 324 bp. Cycles were 35 cycles using a 94 °C melt temperature (30 s), hybridization at 51 °C (60 s), and extension at 72 °C (60 s).

PSEN1 5’**.**AATAGAGAACGGCAGGAGCA.3’ transgene forward and 5’.GCCATGAGGGCACTAATCAT.3’ transgene reverse with internal control primers as per APP. PSEN1 amplicon = 608 bp. 35 cycles using a 94 °C annealing temperature (30 s), hybridization at 54 °C (60 s), and extension at 72 °C (60 s).

*CTR—*the presence of the deleted allele of the mouse CTR locus was identified by PCR using with a 5’ exon 11 primer 5’.GCTGGCTGAGTGCAGAAA.3’ and a 3’ primer CTRR8 5’.CGGTGAGTAATGAATGAAGTGAA.3’. Using Elongase Taq polymerase (Invitrogen 10,480–028), PCR was performed for 35 cycles using a 94 °C annealing temperature (30 s), hybridization at 58 °C (30 s), and extension at 68 °C (90 s).

*Pde6b*—mice entered for behavioral testing were also genotyped for *rd1* to exclude any homozygotes with retinal degeneration caused by the proviral element insertion into the Pde6b gene. Primers were 5’.AAGCTAGCTGCAGTAACGCCATTT.3’ mutant allele and 5.ACCTGCATGTGAACCCAGTATTCTATC.3’ wild-type allele and using 5’.CTACAGCCCCTCTCCAAGGTTTATAG.3’ as a common primer. The mutant allele amplicon is 560 bp and the wild-type allele is 240 bp. PCR with Taq polymerase (Invitrogen) was performed with a 94 °C melt step (300 s) followed by 35 cycles using a 94 °C melt temperature (30 s), hybridization at 65 °C (20 s), and extension at 72 °C (90 s).

All primers were used at 0.4 μM of each final concentration and PCR reactions were performed in 25 μl volumes with 200 ng of genomic DNA. After polymerization cycles, reactions were incubated at 120 s at 72 °C for chain completion before electrophoretic analysis.

### Behavioral Testing

#### Morris Water Maze (MWM)

The MWM apparatus (Harvard Apparatus) consisted of 2-m circular blue plastic pool filled with water (24–25 °C), which was rendered opaque by the addition of nontoxic white paint. An escape platform (20 cm in diameter) was submerged 0.5 cm under the water level. Dark posters, different in shape (one per wall), provided distant landmarks. The behavior of a mouse was recorded by a video camera connected to a video tracking system (HVS Image 2100, HVS Image, Buckingham, UK). The pool was surrounded by a white curtain, and a mouse was released facing the wall at points (N, E, S, W) which were chosen semi-randomly. The mice were trained for 3 days (4 trials per day) to find a submerged platform located in the center of the NE quadrant of the pool (target quadrant, TQ). The trial ended when a mouse found and climbed onto the platform within 120 s. If the mouse failed to find the platform, it was guided to the platform by an experimenter. After a 10-s post-trial time on the platform, the mouse was placed in a holding cage to dry. Mice were tested with inter-trial interval of 50 min. Memory was evaluated in probe trial, administered on day 4 as the first trial of the day. During probe trial, the platform was removed from the pool. Memory for the platform location was expressed as the percent of time spent in TQ.

### Slice Preparation and Electrophysiology

**Fig. 1:**
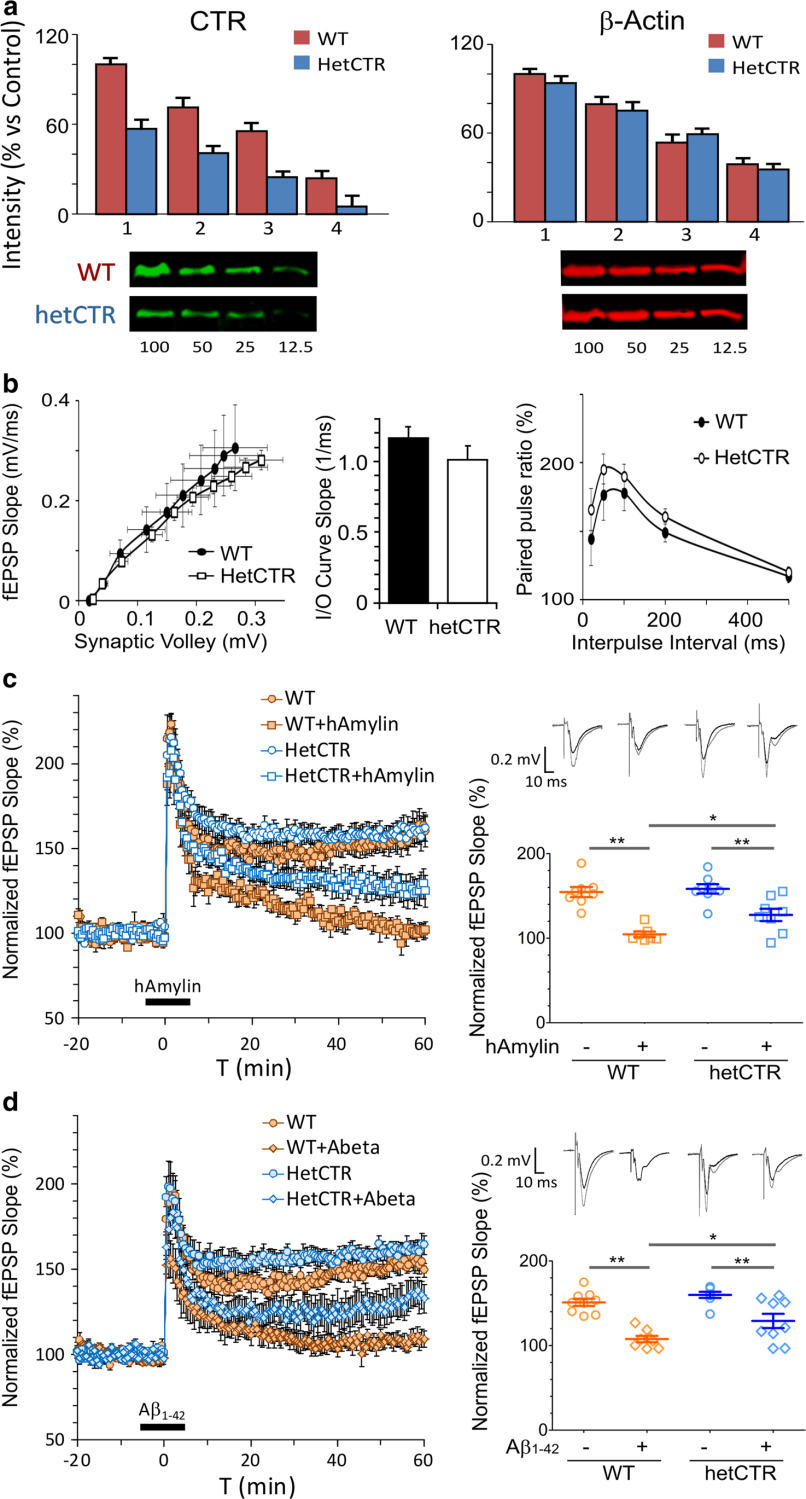
Amylin and amyloid beta (Aβ) modulation of hippocampal long-term potentiation (LTP) in CTR depleted mice. **a** Western blot shows CTR protein expression in HetCTR mouse brain which is reduced to approximately 50% level of that for WT control littermate mice. Histograms (top) and representative western blots (below) show levels of CTR and the loading control β-actin following serial dilutions of whole brain protein from HetCTR and WT mice (*n* = 3 each group). **b** Right and middle panels showing input–output (I/O) curves that do not demonstrate significant change in basal synaptic transmission between HetCTR (*n* = 6, one slice per mouse) and WT mice (*n* = 5, one slice per mouse). Left panel: paired-pulse facilitation across different interstimulus intervals (20–500 ms) in HetCTR (*n* = 6 one slice per mouse) and WT mice (*n* = 5, one slice per mouse). **c, d** LTP traces and scatter plots show that there was no difference between WT and HetCTR mice. In WT mice, applications of hAmylin (50 nM) or Aβ _1–42_ (50 nM) induced a reduction of LTP. However, hemizygosity for the CTR locus significantly reduced this hAmylin (50 nM)- or Aβ_1-42_ (50 nM)-induced LTP inhibition (*n* = 8–9, one slice per mouse for each group, *p < 0.05, **p < 0.01; one-way ANOVA followed Tukey’s test). All results shown are mean ± SEM

Brains were quickly removed from mice following decapitation and placed in a cold artificial cerebral spinal fluid (aCSF) on a vibratome chamber, and transverse sections cut through the hippocampus. The aCSF contained (in millimolar) 124 NaCl, 3 KCl, 2.4 CaCl_2_, 2 MgCl_2_, 1.25 NaH_2_PO_4_, 26 NaHCO_3_, and 10 D-glucose and was equilibrated with 95% O_2_ and 5% CO_2_. Hippocampal slices (400-μm thick) were maintained in aCSF-filled holding chamber at 28 °C for at least 1 h and individually transferred to the submerged glass bottom recording chamber, which was constantly perfused with aCSF (2 ml/min) at 30 °C. Field excitatory postsynaptic potential (fEPSP) was recorded with a metallic (Pt/Ir) electrode (FHC, Bowdoin, ME) from the stratum radiatum layer of cornu ammonis 1 (CA1) region of the hippocampus area, and the Schaffer collateral afferents were stimulated with 100-μs test pulses via a bipolar cluster electrode (FHC; Fig. 1). To evaluate basal synaptic transmission, we applied different stimulation strengths (50 to 275 μA in increments of 25 μA) and plotted fEPSP slopes versus amplitudes of the presynaptic fiber volleys in order to compare the slope of input/output (I/O) curves of fEPSP. In the following experiments, stimulus current was adjusted so that fEPSP stabilized at 40–50% of maximum. To test paired-pulse facilitation, we measured the percentage increase in the slope of fEPSP relative to the first one with different inter-pulse intervals (20–500 ms). For long-term potentiation (LTP) experiments, test pulses were delivered to Schaffer collaterals once every 30 s. LTP was induced by 3 times-theta-burst stimulation (3-TBS) protocol (each burst consisted of four pulses at 100 Hz with a 200-ms inter-burst interval). Before 3-TBS or drug application, the responses were monitored for at least 10 min to ensure a stable baseline of fEPSP. To determine whether the magnitude of LTP differed significantly between groups, average responses during the last 20-min block of recordings (40–60 min after TBS) were compared. All drugs and chemicals were applied directly to the slice via bath perfusion, which allowed for a complete exchange of the perfusate in less than a minute and a half.

### Thioflavin S Amyloid Plaque Staining

After completion of treatments, all mice were sacrificed with an overdose of isoflurane anesthetic, perfused transcardially with normal saline using a syringe infusion pump (Harvard Apparatus) at 5 min/min rate for 5 min. For brain vessel tracing, mice were perfused for another 5 min with Evans Blue (Sigma) solution (1%) in PBS. The brains were dissected, right hemisphere was frozen for biochemical analysis (western blot), and the left hemisphere was fixed with 4% paraformaldehyde-PBS for 24 h at 4 °C. These brain tissues were further processed with modified CLARITY protocol (http://www.chunglabresources.com/clarity/). Briefly, the fixed brain tissue was transferred to hydrogel monomer solution (4% polyacrylamide in PBS) at 4 °C for 24 h, and subsequently to a 24-well plate, merged in fresh hydrogel solution and the tissue brought to 37 °C till formation of the gel. The sagittal slices (400 μm) were cut on an HR2 Slicer (Sigman Electronic, Germany) and cleared with 8% SDS in PBS for 24 h, followed by 0.3% Triton X-100 in PBS for 24 h. A modified thioflavin S staining was used for detecting Aβ plaques. Briefly, the brain sections were rinsed with distilled water, incubated with thioflavin S (0.0125% in 50% ethanol) solution for 5 min, and then washed with 50% ethanol and water. The brain slices were further incubated with DAPI (Invitrogen) in PBS solution for 5 min. The stained clear slices were mounted on a glass slide using Dow Corning high vacuum grease that surrounds to cylinder shapes of a thickness slightly more than the thickness of slice. Images were captured using fluorescence microscopy (Axioplan-2, Carl Zeiss Ltd). Amyloid plaque size and area were analyzed with Image J software.

### Immunofluorescence for Mouse Brain Slices

The cleared brain slices were first blocked in 2% BSA + 10% goat serum for 4 h. The brain slices were then exposed to primary antibodies for 24 h at 4 °C, followed by washing with PBS and exposure to secondary antibodies for 4 h.

### Human Endothelial Cell Culture and Stain

Human dermal microvascular endothelial (HMEC-1, ATCC® CRL-3243™) cells were grown in MCDB131 medium (Life Technologies) with 10% FBS. For amyloid plaque formation, HMEC-1 cells were plated at 2000 cells per well in an 8-well chamber culture slide incubated with 1 µM Aß_1-42 _for 24 h. Cells were washed with room temperature PBS three times (3 ×), fixed with 4% paraformaldehyde-PBS for 10 min, and washed 3 × with DPBS. Cells were incubated for 1 h in blocking solution (1% BSA containing 5% goat serum). Primary antibodies (6E10, CTR, RAMP3) were applied 2 h at room temperature. Cells were washed 3 × in PBS, incubated with secondary antibodies (Alexa Fluor) with DAPI for 1 h at room temperature, and washed 3 × for 10 min in PBS. Secondary antibodies were used at 1:500 dilution (all Alexa Fluor antibodies from Thermo Fisher). Fluorescent imaging was performed on an Axioplan 2 fluorescence microscopy (Carl Zeiss Ltd).

### Western Blot

Frozen brain tissues or cultured cells were homogenized in cold RIPA buffer with protease inhibitors and proteins were quantified with BCA assay (BioRad, Mississauga, ON, Canada). Proteins were loaded at 50 μg per lane on a 12% polyacrylamide gel. Proteins were transferred to nitrocellulose membrane and then blocked with Odyssey blocking buffer. Blots were further incubated with primary antibodies overnight at 4 °C on a shaker. IRDye 800CW goat anti-rabbit and IRDye 680CW goat anti-mouse (Li-Cor, 1:10,000) were used as secondary antibodies. Blots were imaged using Odyssey image system (Li-Cor).

### Experimental Design and Statistical Analysis

All experiments in the current study were performed on age-matched, male and female 5XFAD, TgCRND8, 5XFAD + HetCTR, TgCRND8 + HetCTR, wild-type (WT), and HetCTR mice. We used independent groups of animals to conduct the electrophysiological experiments and behavioral tests. For electrophysiological experiments, one hippocampal brain slice per mouse was used for each group (*n* = 8–9 mice). Seven to 12 mice per group were used for behavioral tests and investigator was blinded to the groups for the behavioral assessments. Experimental details specific for behavioral testing are included in the “Behavioral Testing” section above. For all biochemical and histology experiments, a minimum of 5 animals per experiment were used. Then the number per group for each individual set of experiments is stated in each figure legend. Unpaired, two-tailed *t* tests were used for comparison between two groups, with *p* ≤ 0.05 considered significant (for LTP and biochemical/histological data). For all comparisons involving multiple variables, one-way ANOVA was performed followed by post hoc test for multiple comparisons using *p* ≤ 0.05 for significance (for LTP and MWM data). Bar graphs have been included to show the appropriate statistical information (mean ± SEM). All statistical analyses were performed using Prism (GraphPad Prism 5, San Diego, CA).

## Results

### Synaptic Plasticity in the Hippocampal Slices from HetCTR (AMY/CTR Depleted) Mice

Depletion of CTR in HetCTR mice was first confirmed with western blot analysis (Fig. 1a). In HetCTR mice, CTR protein expression is reduced compared to control WT mice following serial dilutions. We next conducted a systematic evaluation of changes in hippocampal synaptic functions in HetCTR mice at 3–4 months of age. In hippocampal slices from ≤ 4-month-old HetCTR mice, input/output (I/O) curves of fEPSPs at Schaffer collateral-CA1 synapses in response to different stimulation strengths were not significantly different from those of WT controls (Fig. 1b). Thus, the basal synaptic transmission as assessed by the average slope of I/O curves was not significantly different in WT and HetCTR mice at 4 months of age. We also tested paired-pulse facilitation, which represents a short-term form of synaptic plasticity reflecting presynaptic release probability of the neurotransmitter. Paired-pulse facilitation was indistinguishable between HetCTR mice and WT controls at 4 months of age (Fig. 1b). Therefore, short-lived presynaptic plasticity was normal in HetCTR mice.

### Human Amylin and Aβ Actions on Hippocampal LTP in HetCTR Mice

No difference in LTP at hippocampal Schaffer collateral-CA1 synapses was identified between HetCTR and WT controls at 4 months of age (Fig. 1c and d). However, exposure of the hippocampal slices from WT mice to either hAmylin (50 nM) or soluble oligomeric Aβ_1-42_ (50 nM) significantly depressed the LTP induced by application of 3-TBS protocol at the CA1 region (*p* < 0.01). Similarly, in HetCTR mice, hAmylin (50 nM) or soluble oligomeric Aβ_1-42_ (50 nM) significantly depressed the LTP (*p* < 0.01). Applications of hAmylin or Aβ_1-42_ induced a significantly lesser depression of LTP in HetCTR versus WT mice (*p* < 0.05). In all experiments, there were *n* = 8–9 slices for each group with one slice per mouse.

### Effects of AMY/CTR Knock Down in TgCRND8 Mice on Hippocampal Long-Term Potentiation

**Fig. 2:**
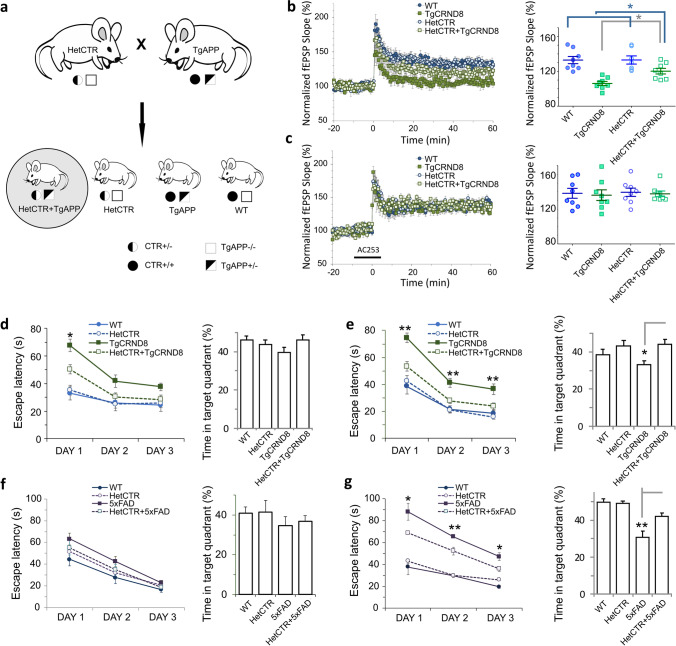
AMY/CTR depletion improves cognitive function in two mouse models of AD. **a** Schematic for generating AD mice hemizygous for the AMY/CTR locus. Two different mouse models expressing human APP (TgCRND8 or 5xFAD) were used for generation of AMY/CTR compound Tg/hemizygous mice. In the schematic shown here, TgAPP refers to either TgCRND8 or 5XFAD strain of mice. **b** LTP traces (left) and scatter plots (right) show that there is a significant LTP reduction in TgCRND8 mice compared to WT mice; depletion of AMY/CTR receptor in TgCRND8 mice significantly improves LTP (HetCTR + TgCRND8 vs Tg CRND8; *n* = 8 for each group, one slice per mouse). No differences between WT (*n* = 8, one slice per mouse) and HetCTR mice (*n* = 7, one slice per mouse) were observed. *p < 0.05, **p < 0.01; one-way ANOVA followed Tukey’s test. **c** Application of AC253 (250 nM), an AMY receptor antagonist, reverses the LTP reductions in both the HetCTR + TgCRND8 and the TgCRND8 mice (*n* = 8 mice for each of the four groups, one slice per mouse). **d, e** Data from Morris water maze (MWM) and probe tests for WT (*n* = 8 mice), HetCTR (*n* = 9 mice), TgCRND8 (*n* = 9 mice), and HetCTR + TgCRND8 (*n* = 12 mice) groups of mice (**d**) at 6 m (early cognitive function impairment for AD mice) and (**e**) at 9 m (significant cognitive impairment) of age (**e**). At 6 months of age, no statistically significant differences in MWM escape latencies or quadrant preference are observed among the four groups. However, at 9 months of age, there is a significant improvement in escape latencies and quadrant preference in 9-month-old HetCTR + TgCRND8 mice compared to their age-matched TgCRND8 littermates. **f, g** Data from MWM and probe test from WT (*n* = 7 mice), HetCTR (*n* = 9 mice), 5xFAD (*n* = 9 mice), and HetCTR + 5xFAD (*n* = 10 mice) groups of mice (**f)** at 3 m (early cognitive function impairment for AD mice) and (**g**) at 8 m (significant cognitive impairment) of age. Similar age-dependent improvement in escape latencies and quadrant preference is seen for HetCTR + 5XFAD mice compared to age-matched 5XFAD mice. For experimental data shown in **b–g**, *p < 0.05, **p < 0.01; one-way ANOVA followed Tukey’s test

A cross-breeding scheme for HetCTR and TgCRND8 mice (Fig. 2a) yielded compound Tg mice (HetCTR + TgCRND8) at the expected 25% frequency. Hemizygosity for the AMY/CTR locus did not alter body weight or blood glucose levels across four groups of age-matched mice (Suppl. Figure 1). We compared the LTP responses in 8 to 12 month-old HetCTR + TgCRND8 compound mice to those obtained from WT, HetCTR and TgCRND8 mice (Suppl. Figure 2). We also examined the LTP responses obtained from each set of mice in the presence of the amylin receptor, AC253.

A comparison of the normalized slope of fEPSP during 40–60 min after induction between the four groups revealed that LTP at hippocampal CA1 synapses was markedly reduced in TgCRND8 mice compared to that observed in age-matched WT and HetCTR littermate mice (*p* < 0.01, Fig. 2b). However, in the HetCTR + TgCRND8 mice, the LTP deficit observed was partially restored to the levels closer to those observed in age-matched WT or HetCTR mice (*p* < 0.05). Together, these results indicate that impairment of hippocampal LTP in TgCRND8 mice was partially rescued by 50% depletion of AMY/CTR receptor genes.

We have previously shown that LTP levels at hippocampal Schaffer collateral-CA1 synapses in TgCRND8 mice can be improved in the presence of the peptidergic amylin receptor antagonist, AC253 (NH_2_-LGRLSQELHRLQTYPRTNTGSNTY-COOH) [5]. Pre-application of AC253 (250 nM) to hippocampal slices for 5 min before and after 3-TBS did not affect basal synaptic transmission or the LTP during 40–60 min after its induction in either WT or HetCTR control mice. LTP levels in HetCTR + TgCRND8 mice were improved compared to the reduced LTP that is observed for TgCRND8 mice. Application of the amylin receptor antagonist, AC253, further elevated the LTP recorded from HetCTR + TgCRND8 mice to levels that were comparable to age-matched WT littermate mice (Fig. 2c). In all above experiments, there were *n* = 8–9 slices for each group with one slice per mouse.

### Spatial Memory in AD Mouse Models with AMY/CTR Knock Down

In order to determine whether the AMY/CTR knock down by 50% can prevent or delay spatial learning and memory deficits in AD mice, we used two different aforementioned transgenic mouse models, namely TgCRND8 and 5xFAD mice.

In TgCRND8 mouse model of early-onset AD, we used Morris water maze (MWM) as a behavioral test for spatial memory at two different time points, at 6 months and 9 months of age. At the age of 6 months, there was no difference seen in the escape latency to locate the hidden platform between WT and HetCTR mice (Fig. 2d). TgCRND8 mice demonstrated a significant impairment in performing the same task when compared to WT and HetCTR control mice (*p* < 0.001) However, the performance of HetCTR + TgCRND8 compound mice was superior to that of age-matched TgCRND8 mice (*p* < 0.05). During the probe trial, no differences in total exploration time spent in quadrant containing the target platform were observed among the four experimental mice groups, indicating no significant impairment in memory retention across the groups at 6 months of age (Fig. 2d). At 9 months of age, the difference in MWM performance of HetCTR + TgCRND8 mice versus TgCRND8 mice was greater with the latencies to locate the hidden platform significantly longer for the latter group across all days of testing (*p* < 0.01). On the other hand, there was no significant difference in performance between TgCRND8-HetCTR mice and WT or HetCTR mice. During the probe trial, there was, at this time point, a significant increase in total exploration time spent in the target platform quadrant for the HetCTR + TgCRND8 compared to TgCRND8 mice (*p* < 0.05, Fig. 2e), indicating improved memory retention in AMY/CTR depleted TgCRND8 AD mice.

In a similar manner, we used MWM as a test of spatial memory in 5xFAD mice at 3 and 8 months of age. At the age of 3 months, there was no significant difference seen in the escape latency to locate the hidden platform between four experimental mice groups (Fig. 2f). However, at the age of 8 months, the latency to locate the hidden platform for 5xFAD mice was significantly longer than that for HetCTR + 5xFAD (*p* < 0.01) and either WT or HetCTR control mice (*p* < 0.001) (Fig. 2f). Additionally, during the probe trial, there was significant increase in total exploration time spent in the target platform-containing quadrant for WT, HetCTR control mice (*p* < 0.001) or the HetCTR + 5xFAD mice (*p* < 0.01) compared to 5xFAD mice (Fig. 2g). HetCTR + 5xFAD mice demonstrated an increase time in target platform quadrant compared to 5xFAD mice (Fig. 2g) indicative of improved memory retention in AMY/CTR depleted 5xFAD mice. In all above experiments, there were *n* = 7–12 for each group as detailed for each genotype in the figure legend.

### Amyloid Pathology in TgCRND8 AD Mice with AMY/CTR Deficiency

**Fig. 3:**
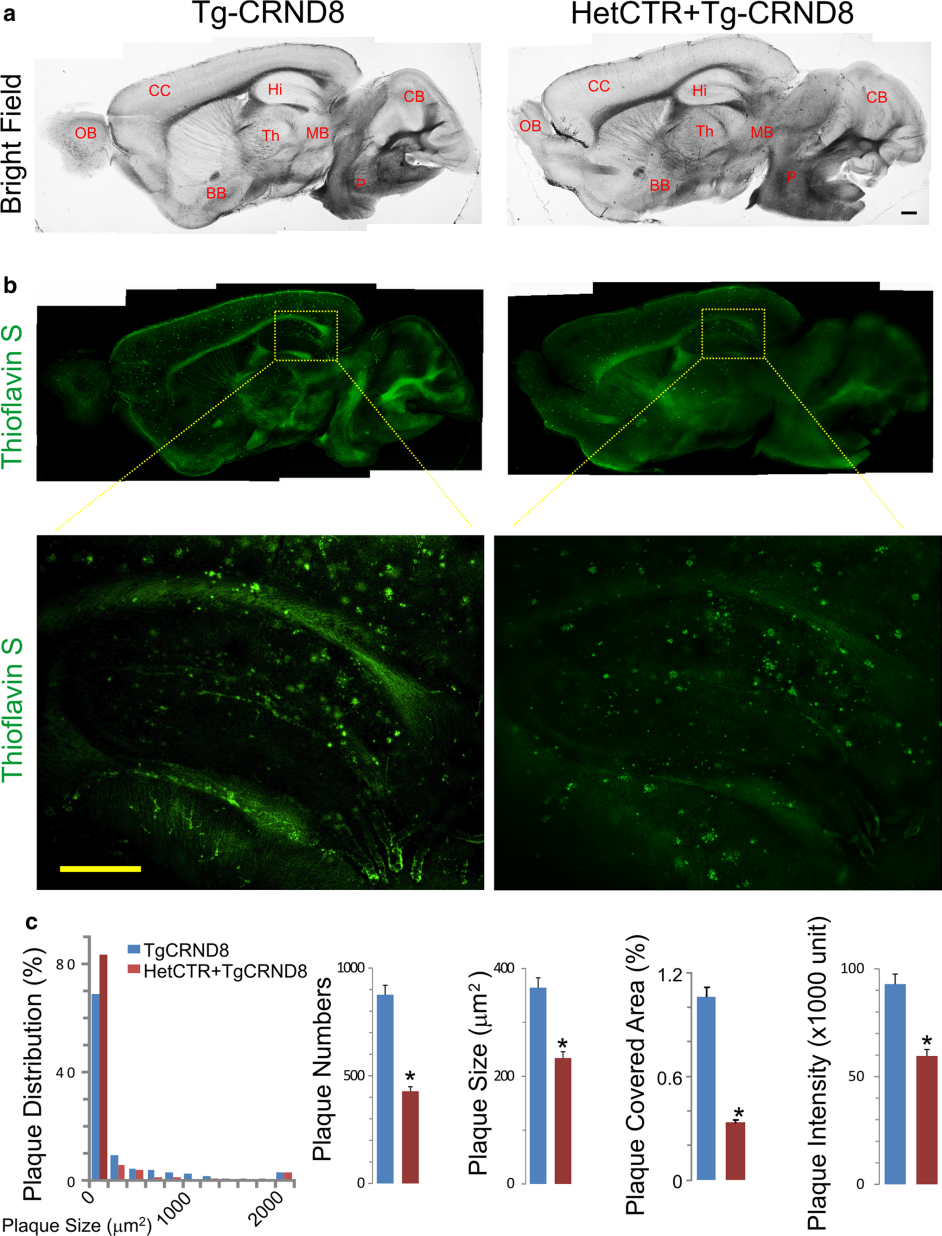
AMY/CTR depletion reduced amyloid pathology in AD mice. **a** Bright field sagittal sections from 8-month-old TgCRND8 and HetCTR + TgCRND8 mice. Abbreviations: OB, olfactory bulb; CC, cerebral cortex; Hi, hippocampus; BB, basal forebrain; Th, thalamus; MB, midbrain; CB, cerebellum; P, pons. **b** Brain sections stained for thioflavin S, showing the presence of amyloid plaques in an 8-month-old HetCTR + TgCRND8 mice compared to TgCRND8 mouse. Boxed areas in these sections are shown at higher magnification in the lower panels and show a reduction in amyloid plaques within the hippocampal region of HetCTR + TgCRND8 compared to TgCRND8 mice. **c** Quantification of plaque numbers, plaque sizes, plaque covered area, and plaque fluorescent intensity in the hippocampal region of TgCRND8 and HetCTR + TgCRND8 mice. Data from *n* = 5 mice in each group: *p < 0.05; *t* test: two sample assuming unequal variances. All results shown are mean ± SEM. Scale bar in lower panel of **b** = 500 µm

At the conclusion of our behavioral experiments on AMY/CTR depleted AD mice, we sought to also examine aspects of AD pathology in these transgenic mice. Thioflavin S staining was used to assess the distribution and morphology of amyloid plaques in brains sections along the sagittal plane at the midline. We observed that the number of amyloid plaques in the hippocampus of HetCTR + TgCRND8 mice was significantly decreased as compared to TgCRND8 mice (Fig. 3a–c); for the plaque size, the average area occupied by the plaques and the plaque intensity were quantified and also found to be reduced in the hippocampus (Fig. 3c; *n* = 5 each group, *p* < 0.05).

### APP and Other Molecular Markers in 5XFAD Mice with AMY/CTR Knock Down

**Fig. 4:**
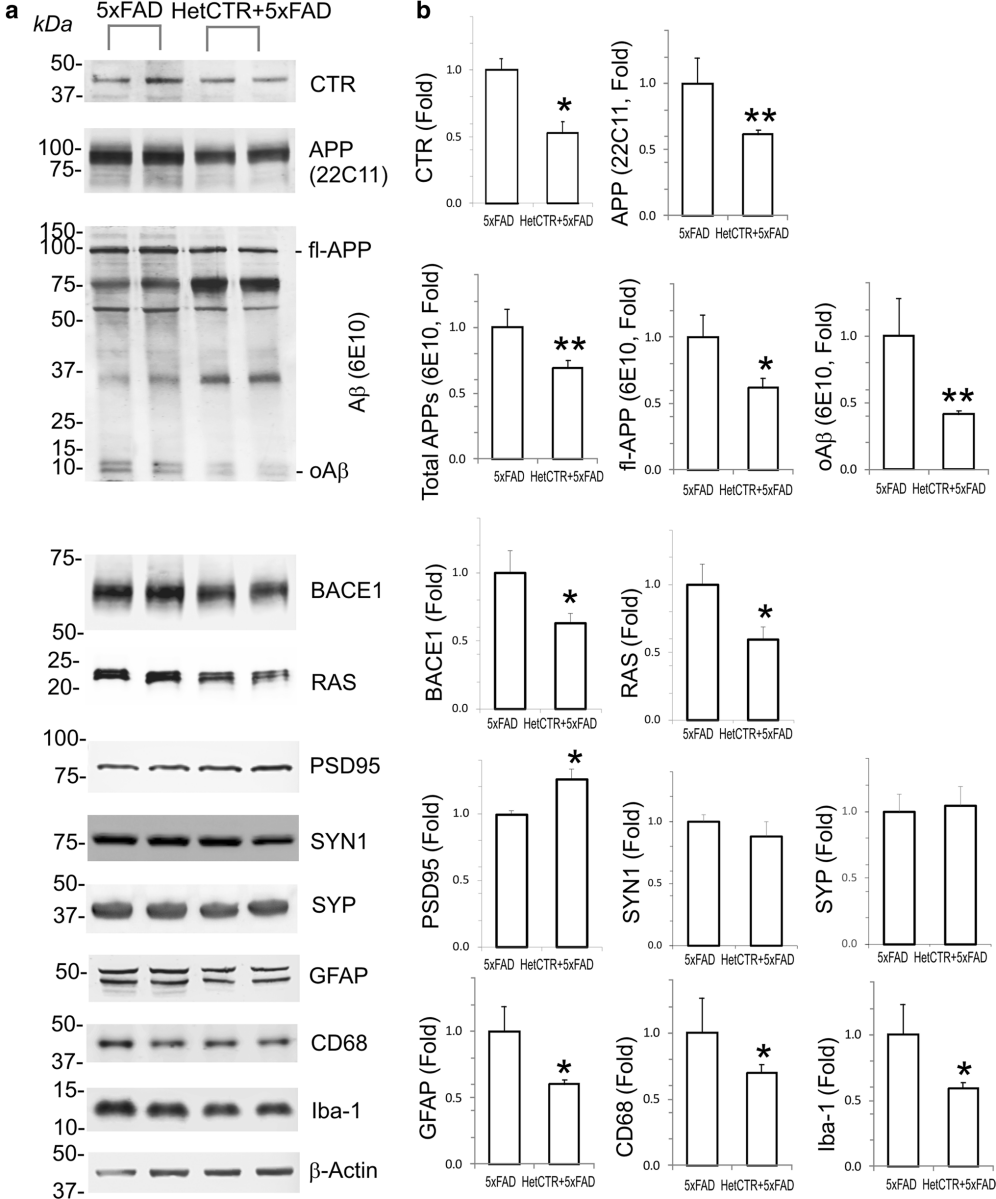
Pathological markers for AD are attenuated in 5xFAD AD mice compared to AD mice hemizygous for the AMY/CTR locus. **a** Western blots from two sets of mice each for 5xFAD and HetCTR + 5xFAD showing calcitonin receptor (CTR), amyloid precursor protein (APP, total and full length), oligomeric amyloid beta (oAβ), β-secretase 1 (BACE1), RAS, PSD95, synapsin 1 (SYN1), synaptophysin (SYP), Iba-1, CD68, GFAP, and β-actin (loading control). **b** Histograms of data showing quantification of western blots from 5xFAD/5xFAD-HetCTR brain tissues. Hemizygosity for the AMY/CTR locus in 5XFAD mice (HetCTR + 5xFAD) in addition to demonstrating depletion of CTR revealed reduced APP, including total APP, full-length APP, APP-22C11, oAβ, BACE1, and RAS. Postsynaptic marker (PSD95) was increased in HetCTR + 5XFAD mice compared to 5XFAD mice but no change observed in presynaptic markers SYN1 and SYP. In HetCTR + 5xFAD mice, a reduction of reactive astrocytes (GFAP) and activated microglia (Iba-1 and CD68) was also observed. Data from *n* = 5 mice for each group; *p < 0.05, **p < 0.01, *t* test: two sample assuming unequal variances. All results shown are mean ± SEM

β- and γ-secretase enzymes are two key elements of the amyloidogenic pathway for APP processing that is associated with the production of Aβ. We therefore investigated whether APP processing could also be affected in 5XFAD strain of AD mice with reduced AMY/CTR expression and to this end measured total APP, full-length APP, β-secretase 1 (BACE1), and soluble oligomeric fraction of Aβ42 in lysates obtained from hippocampal and cortical tissue from 5XFAD and HetCTR + 5XFAD mice (*n* = 5 for each group). Protein levels of total APP, full-length APP, BACE1, and soluble oligomeric Aβ in the brains of HetCTR + 5XFAD mice were significantly reduced in comparison to those from 5XFAD mice (Fig. 4a, b; Suppl. Figure 3; *p* < 0.05).

No changes in either synapsin 1 or synaptophysin (presynaptic markers) were observed between HetCTR + 5XFAD mice and 5XFAD mice that is consistent with the paired-pulse electrophysiological data showing no presynaptic plasticity. However, PSD95 (postsynaptic marker) levels in HetCTR + 5XFAD mice were increased compared to 5XFAD mice. Downstream signaling protein, RAS, a GTPase that is linked to amylin receptor activation, is reduced in HetCTR + 5XFAD mice compared to 5XFAD mice (Fig. 4b; Suppl. Figure 3; *p* < 0.05).

We also examined microglial (CD68 and Iba-1) and astrocytic (GFAP) markers in brain lysates from the two strains of transgenic mice. Protein levels of CD68, Iba-1, and GFAP as determined by western blot analysis and quantified using Image J analysis also were significantly lower in HetCTR + 5XFAD mice compared to 5XFAD mice (Fig. 4b; Suppl. Figure 3; *p* < 0.05).

### Involvement of AMY/CTR Receptor in Vascular Amyloid Pathology

**Fig. 5:**
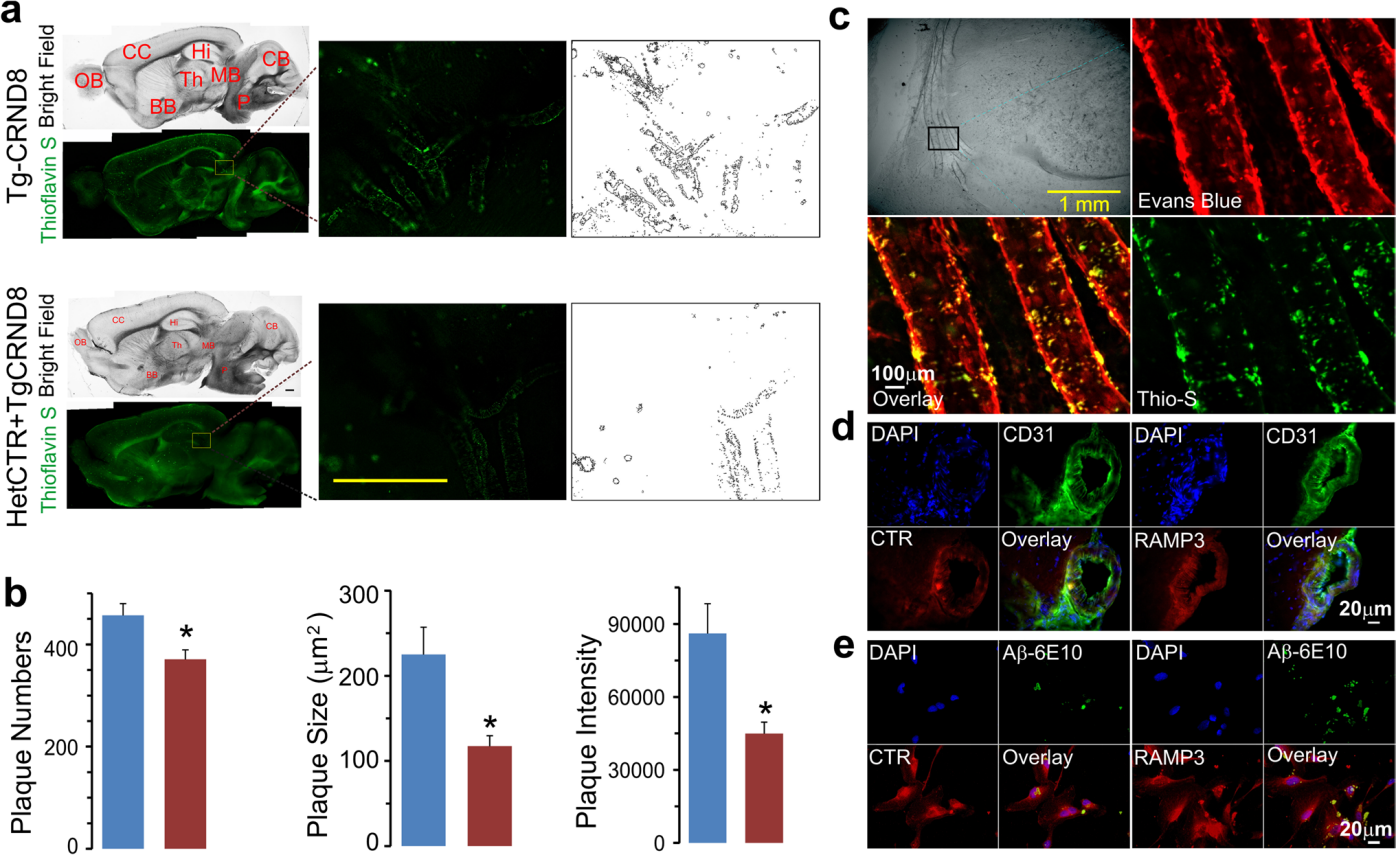
Vascular amyloid pathology in AMY/CTR depleted AD mice. **a** Left, bright field and thioflavin S stained sagittal brain sections from TgCRND8 and HetCTR + TgCRND8 mice. Right, higher magnification of sections showing amyloid deposition within the cerebral vasculature (left) and Image J generated profiles of amyloid-positive blood vessels (right). Hemizygosity for AMY/CTR results in a reduction in vascular amyloid pathology in AD mice. Scale bar = 500 µm. **b** Histograms depict quantification of amyloid plaque number, size, and intensity using Image J analysis of the thioflavin S-positive blood vessels. Data are from 5 mice in each group; *p < 0.05, *t* test: two sample assuming unequal variances. All results shown are mean ± SEM. **c** Bright field image from a TgCRND8 mouse showing cerebral blood vessels that are stained with Evans blue and shown from the boxed area at a higher magnification in the right panel. Counterstaining with thioflavin S of this section reveals co-localization of the amyloid within vessel walls. **d** Sections through the cerebral vasculature show immunohistochemical co-localization of CTR and RAMP3, components of the amylin receptor, on endothelial cells (identified with CD31 staining). **e** The CTR and RAMP3 are present on human microendothelial cells (HMEC-1). After exposure of HMEC-1 cells to Aβ_1-42_ (0.5 µM) for 24 h, there is an accumulation of amyloid on and in the vicinity of cells that are positive for CTR and RAMP3

Thioflavin S staining method was used to assess the distribution and morphology of amyloid plaques within cerebral blood vessels from the cortex sectioned along the sagittal plane. Amyloid pathology was readily visualized within the cerebral vasculature of TgCRND8 mice, but appeared reduced in HetCTR + TgCRND8 mice (Fig. 5a , *n* = 5 for each group). The number of amyloid plaques, plaque size, and their intensity were quantified and were observed to be reduced within the blood vessels from HetCTR + TgCRND8 mice compared to those from TgCRND8 mice (Fig. 5b; *p* < 0.05). In addition to the abundant vascular amyloid pathology (Fig. 5c), the presence of AMY/CTR receptor expression within the walls of the cerebral blood vessels of TgCRND8 mice was visualized using CTR and RAMP3 antibodies (Fig. 5d). AMY/CTR receptor was present on endothelial cells (HMEC-1) and Aβ plaque-like deposits were observed on HMEC-1 cells expressing CTR and RAMP3 heterodimeric components of the amylin receptor (Fig. 5e).

## Discussion

### Amylin Receptor Action and AD Pathogenesis

The amylin receptor, a class B GPCR, is viewed as a plausible therapeutic target for AD based upon observations that modulation of this receptor by either synthetic amylin analogs or amylin antagonists confers improvement in spatial memory and learning [6–8, 16, 17]. In order to resolve the dichotomy of whether it is activation or blockade of the amylin receptor that holds promise as a therapeutic strategy, we used a genetic approach consisting of a deletion within one copy of an endogenous chromosomal locus. In this study, we show for the first time that a partial depletion of a critical heterodimeric component of the amylin receptor, CTR, superimposed on expression of human APP695 with two familial AD mutations, results in improvement of memory-related responses in the resulting compound Tg mice. In hippocampal brain slices from these HetCTR mice, the impairment of hippocampal LTP induced by exposure to human amylin or Aβ is ameliorated by approximately 50% compared to that in wild-type (WT) littermate controls. The attenuation of Aβ or human amylin-evoked LTP responses thus proved proportionate to the reduction of CTR gene copy number (and hence the amylin receptor protein) in HetCTR mice. Additionally, the reduction of LTP responses normally observed in human APP-expressing TgCRND8 mice is significantly improved in HetCTR + TgCRND8 compound mice. HetCTR + 5XFAD mice also demonstrate an increase in the postsynaptic marker, PSD95, compared to 5XFAD mice. These electrophysiological observations suggest that a reduction in the amylin receptor leads to a significant improvement in an electrophysiological response that is widely accepted as a cellular surrogate of memory. Furthermore, in the HetCTR + TgCRND8 and HetCTR + 5xFAD mice, we observed a significant improvement in spatial memory compared to the two strains of AD mice expressing mutant FAD forms of human APP695 in the context of a full complement of CTR expression (i.e., in animals that were homozygous WT for the CTR locus).

Deposition and fibrillation of human Aβ in both the TgCRND8 and 5xFAD mice is an age-dependent process that is important in AD pathogenesis and matched by progressive deficits in memory performance and cellular surrogates of memory [9, 13, 14, 18, 19]. The effects of a substantial reduction in a putative target for the deleterious actions of the Aβ, namely AMY/CTR, would be expected to become more apparent with time. Indeed, the observed improvement in spatial memory in HetCTR + TgCRND8 or HetCTR5 + FAD mice compared to TgCRND8 or 5xFAD controls increased as the mice aged. The improvements in spatial memory and learning observed here are thus remarkably similar to those observed following administration of amylin receptor antagonists to AD mice and support the notion that it is blockade or depletion of the target receptor, AMY/CTR, that confers improvement in cognitive function in AD mice [6, 20]. Conversely, the concept that stimulation of AMY/CTR produces an anti-pathogenic effect would predict that depletion of AMY/CTR would reduce the observed benefits and hence accentuate pathogenesis, a result opposite to the ameliorated outcome measures defined here [8, 9, 16].

Besides consideration of LTP and behavioral performance, examination of the brains of HetCTR + TgCRND8 mice also revealed a significant decrease in several markers of AD pathology-amyloid plaque burden, BACE1, and soluble oligomeric Aβ. The mechanism(s) whereby these unique markers of amyloid pathology in AD are attenuated are not yet clear. It is possible that the driver of the age-dependent amyloid pathology is in some unknown ways linked to the activity of AMY/CTR since we have previously observed a parallel age-dependent increase in CTR and Aβ in the hippocampal and cortical brain regions of transgenic AD mice [4]. A robust influence of altered AMY/CTR signaling upon Aβ-directed pathogenesis is supported by improvements in memory and pathology scored in two independent lines of APP695 transgenic mice that have both different genetic backgrounds and different tempos of AD-related changes [13, 14]. Moreover, a recent study revealed that amylin and its synthetic analog, pramlintide, when administered to AD mice, result in *increased* Aβ production via upregulation of APP and its processing enzyme, γ-secretase [17].

Additional explanations for a reduction in amyloid pathology likely involve roles for glia and the brain vasculature, both of which are invested with amylin receptors [21, 22]. Since AMY/CTR complexes are localized on microglia, depletion of these target receptors for Aβ could attenuate the amyloid-induced neuroinflammation, including the cytokine release that is known to upregulate APP processing and thereby increase the rate of Aβ generation [23]. In the present study, we also identified a decrease in markers for activated microglia (and reactive astrocytes) in AMY/CTR depleted AD mice compared to AD mice. Thus, a reduction of a putative receptor target for Aβ on microglia would be expected to result in diminished cytokine release and hence a reduction in APP processing. Indeed, we also observed a reduction in the APP processing enzyme, BACE1, in HETCTR depleted 5XFAD mice compared to 5XFAD mice. These findings would be also consistent with our previous pharmacological data showing that applications of antagonists of the amylin receptor can attenuate release of cytokines IL-1β and TNF-α in vitro and in the brains of AD mice and a reduction in Aβ production [21].

The amyloid deposition in cerebral vasculature constitutes one leg of a “three-legged stool” for pathologic change, with neuronal/synaptic compromise and neuroinflammation being the other two [21]. The demonstrated presence of the AMY/CTR in brain vasculature, in addition to that on neurons/synapses and microglia, suggests that it may also play a notable role in pathogenesis. In previous work, glial AMY/CTR receptors have been shown to promote Aβ influx into cells and subsequent formation of amyloid plaques [24]. In the vasculature, AMY/CTR receptors are localized on endothelial cells and thus may play a role in amyloid deposition in cerebral vessels. In this study, we observed a significant reduction in amyloid plaque numbers, size, and intensity in the brain vasculature of HetCTR + TgCRND8 mice compared to TgCRND8 mice. Experimentally, application of fluorescently labeled Aβ on cultures of HMEC (endothelial) cells resulted in accumulation of amyloid within and over the cells in a plaque-like manner. Collectively, these observations suggest that activity of AMY/CTR receptors located on endothelial cells of the brain vasculature likely promotes amyloid deposition into vessel walls. Conversely, the reduction of this activity due to receptor depletion in HetCTR + TgCRND8 compound mice reduces amyloid deposition and associated AD pathology at these sites.

### Modulation of the AMY/CTR Axis and Clinical Interventions

Amylin (IAPP) is being increasingly viewed as a “second amyloid” with its identification in AD brains and particularly those AD patients with a history of diabetes mellitus [25, 26]. However, we did not observe any overt systemic effects of AMY/CTR reduction in either HetCTR or HetCTR + 5xFAD compound Tg mice; body weight and blood glucose levels did not change in either of the two strains of aged mice. Lack of these types of side effects is thus promising for adoption of a pharmacological approach aimed at tackling the serious pathological consequences of the two types of amyloid, namely, beta amyloid and amylin.

In summary, using a combination of electrophysiological, behavioral, and anatomical approaches, the results of this study provide evidence that genetic depletion of AMY/CTR receptors in AD mouse models improves hippocampal synaptic plasticity and spatial memory, which is associated with a significant reduction in certain characteristic brain markers that are indicative of AD pathology. From a clinical perspective, the amylin receptor thus represents an attractive target for AD with the potential to improve cognition without substantial adverse effects of this therapeutic strategy.

## Supplementary Information

Below is the link to the electronic supplementary material.

Supplementary file1 (PDF 504 KB)

## Data Availability

All data generated or analyzed during this study are included in this published article [and its supplementary information files].

## References

[1] Gallardo G, Holtzman DM (2019). Amyloid-β and Tau at the crossroads of Alzheimer's disease. Adv Exp Med Biol.

[2] Hardy J (2017). The discovery of Alzheimer-causing mutations in the APP gene and the formulation of the “amyloid cascade hypothesis”. FEBS J.

[3] Walsh DM, Selkoe DJ (2020). Amyloid β-protein and beyond: the path forward in Alzheimer’s disease. Curr Opin Neurobiol.

[4] Jhamandas JH, Li Z, Westaway D, Yang J, Jassar S, MacTavish D (2011). Actions of β-amyloid protein on human neurons are expressed through the amylin receptor. Am J Pathol.

[5] Kimura R, MacTavish D, Yang J, Westaway D, Jhamandas JH (2012). Beta amyloid-induced depression of hippocampal long-term potentiation is mediated through the amylin receptor. J Neurosci.

[6] Soudy R, Patel A, Fu W, Kaur K, MacTavish D, Westaway D, Davey R, Zajac J (2016). Cyclic AC253, a novel amylin receptor antagonist, improves cognitive deficits in a mouse model of Alzheimer’s disease. Alzheimers Dement (NY).

[7] Fu W, Patel A, Kimura R, Soudy R, Jhamandas JH (2017). Amylin receptor: a potential therapeutic target for Alzheimer’s disease. Trends Mol Med.

[8] Adler BL, Yarchoan M, Hwang HM, Louneva N, Blair JA, Palm R, Smith MA, Lee HG (2014). Neuroprotective effects of the amylin analogue pramlintide on Alzheimer’s disease pathogenesis and cognition. Neurobiol Aging.

[9] Zhu H, Wang X, Wallack M, Li H, Carreras I, Dedeoglu A (2015). Intraperitoneal injection of the pancreatic peptide amylin potently reduces behavioral impairment and brain amyloid pathology in murine models of Alzheimer’s disease. Mol Psychiatry.

[10] Hay DL, Christopoulos G, Christopoulos A, Poyner DR, Sexton PM (2005). Pharmacological discrimination of calcitonin receptor: receptor activity-modifying protein complexes. Mol Pharmacol.

[11] Hay DL, Chen S, Lutz TA, Parkes DG, Roth JD (2015). Amylin: pharmacology, physiology, and clinical potential. Pharmacol Rev.

[12] Davey RA, Turner AG, McManus JF, Chiu WS, Tjahyono F, Moore AJ (2008). Calcitonin receptor plays a physiological role to protect against hypercalcemia in mice. J Bone Miner Res.

[13] Chishti MA (2001). Early-onset amyloid deposition and cognitive deficits in transgenic mice expressing a double mutant form of amyloid precursor protein. J Biol Chem.

[14] Oakley H, Cole SL, Logan S, Maus E, Shao P, Craft J, Guillozet-Bongaarts A, Ohno M (2006). Intraneuronal beta-amyloid aggregates, neurodegeneration, and neuron loss in transgenic mice with five familial Alzheimer’s disease mutations: potential factors in amyloid plaque formation. J Neurosci.

[15] Dahlgren KN, Manelli AM, Stine WB, Baker LK, Krafft GA, LaDu MJ (2002). Oligomeric and fibrillar species of amyloid-beta peptides differentially affect neuronal viability. J Biol Chem.

[16] Zhu XX, Wang E, Wallack M, Na H, Hooker JM, Kowall N, Tao Q, Stein TD (2017). Amylin receptor ligands reduce the pathological cascade of Alzheimer’s disease. Neuropharmacology.

[17] Mousa YM, Abdallah IM, Hwang M, Martin DR, Kaddoumi A (2020). Amylin and pramlintide modulate γ-secretase level and APP processing in lipid rafts [published correction appears in Sci Rep. 2020 Jul;10(1):11096]. Sci Rep.

[18] Kimura R, Devi L, Ohno M (2010). Partial reduction of BACE1 improves synaptic plasticity, recent and remote memories in Alzheimer’s disease transgenic mice. J Neurochem.

[19] Kachooei E, Moosavi-Movahedi AA, Khodagholi F, Mozaffarian F, Sadeghi P, Hadi-Alijanvand H, Ghasemi A, Saboury AA (2014). Inhibition study on insulin fibrillation and cytotoxicity by paclitaxel. J Biochem.

[20] Soudy R, Kimura R, Patel A, Fu W, Kaur K, Westaway D, Yang J, Jhamandas J (2019). Short amylin receptor antagonist peptides improve memory deficits in Alzheimer’s disease mouse model. Sci Rep.

[21] Fu W, Vukojevic V, Patel A, Soudy R, MacTavish D, Westaway D, Kaur K, Goncharuk V (2017). Role of microglial amylin receptors in mediating beta amyloid (Aβ)-induced inflammation. J Neuroinflammation.

[22] Wang E, Zhu H, Wang X, Gower AC, Wallack M, Blusztajn JK, Kowall N, Qiu WQ (2017). Amylin treatment reduces neuroinflammation and ameliorates abnormal patterns of gene expression in the cerebral cortex of an Alzheimer’s disease mouse model. J Alzheimers Dis.

[23] Blasko I, Marx F, Steiner E, Hartmann T, Grubeck-Loebenstein B (1999). TNF alpha plus IFN gamma induce the production of Alzheimer beta-amyloid peptides and decrease the secretion of APPs. FASEB J.

[24] Fu W, Shi D, Westaway D, Jhamandas JH (2015). Bioenergetic mechanisms in astrocytes may contribute to amyloid plaque deposition and toxicity. J Biol Chem.

[25] Jackson K, Barisone GA, Diaz E, Jin LW, DeCarli C, Despa F (2013). Amylin deposition in the brain: a second amyloid in Alzheimer disease?. Ann Neurol.

[26] Martinez-Valbuena I, Valenti-Azcarate R, Amat-Villegas I, Riverol M, Marcilla I, de Andrea CE, Sánchez-Arias JA, Del Mar C-A (2019). Amylin as a potential link between type 2 diabetes and Alzheimer disease. Ann Neurol.

