# Supplementary material for: Genetic Depletion of Amylin/Calcitonin Receptors Improves Memory and Learning in Transgenic Alzheimer’s Disease Mouse Models
Source: Mol Neurobiol. 2021 Jul 27;58(10):5369–82. doi: 10.1007/s12035-021-02490-y (PMC8497456; doi:10.1007/s12035-021-02490-y)
Supplement: Supplementary file 1 — Supplementary file1 (PDF 504 KB) [file 12035_2021_2490_MOESM1_ESM.pdf]

Supplemental Information: Suppl Figs. 1-3 and Suppl Table 1

Genetic depletion of amylin/calcitonin receptors improves memory and learning in transgenic Alzheimer's disease mouse models.

*Aarti Patel[1], *Ryoichi Kimura[1,2], *Wen Fu[1], Rania Soudy[1,6], David MacTavish[1], David Westaway[1,3,4], Jing Yang[1,4], Rachel A Davey[5], Jeffrey D Zajac[5] and Jack H Jhamandas[1,**]

[1]Department of Medicine (Neurology), Neuroscience and Mental Health Institute, University of Alberta, Edmonton, AB, Canada T6G 2S2
[2] Center for Liberal Arts and Sciences, Sanyo-Onoda City University, Yamaguchi 756-0884, Japan.
[3]Department of Biochemistry, University of Alberta, Edmonton, AB, Canada T6G 2H7
[4]Centre for Prions and Protein Folding Diseases, University of Alberta, Edmonton, AB, Canada T6G 2M8
[5]Department of Medicine, Austin Health, University of Melbourne, Heidelberg, Victoria 3074, Australia
[6]Faculty of Pharmacy, Cairo University, Egypt

Suppl Fig. 1

.

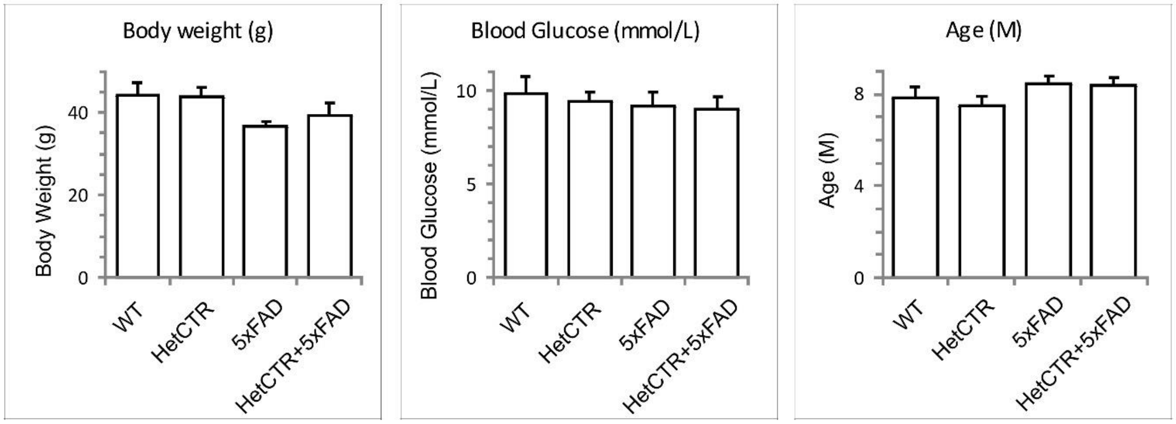

Suppl Fig 1. Hemizygosity for the AMY/CTR locus does not significantly alter parameters of energy metabolism. No significant changes in body weight and glucose levels between the four groups of age-matched littermate mice (WT n=7, HetCTR n=9, 5xFAD n=9 and HetCTR+5xFAD n=10).

Suppl Fig. 2

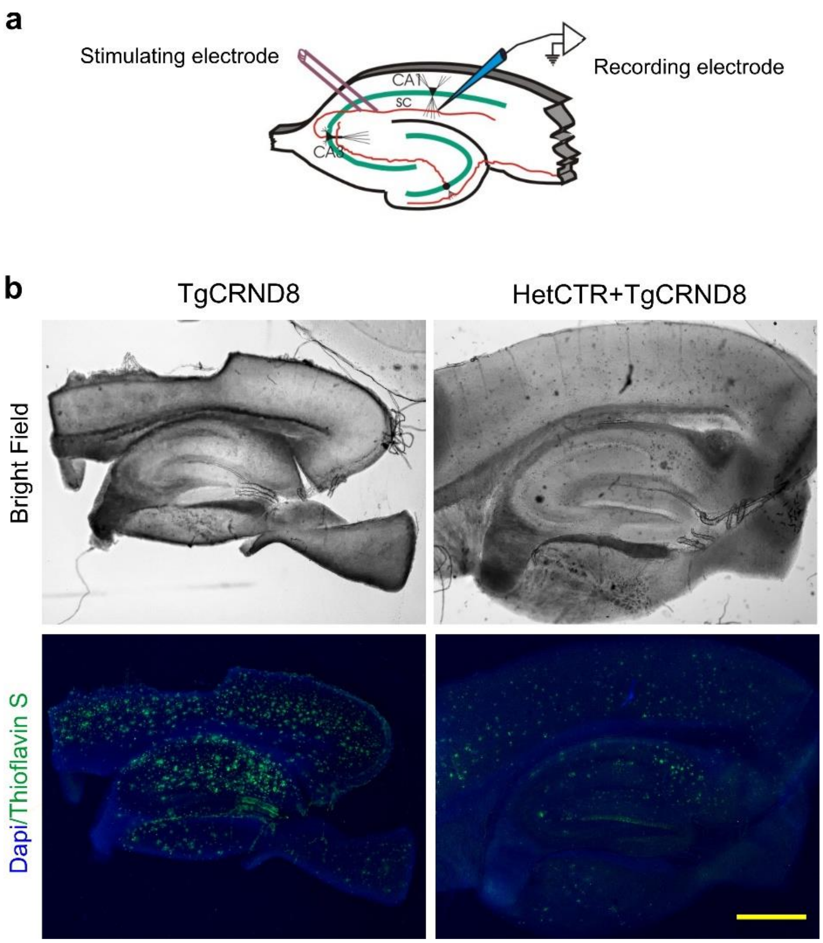

Suppl Fig 2. A, Schematic of hippocampal circuitry for the generation of long term potentiation (LTP). Field excitatory postsynaptic potentials recorded from the CA1 area following stimulation of the Schaeffer collaterals (SC). **B,** Brightfield and thioflavin S stained sections of the hippocampus slices from TgCRND8 and HetCTR+TgCRND8.

Suppl Fig. 3

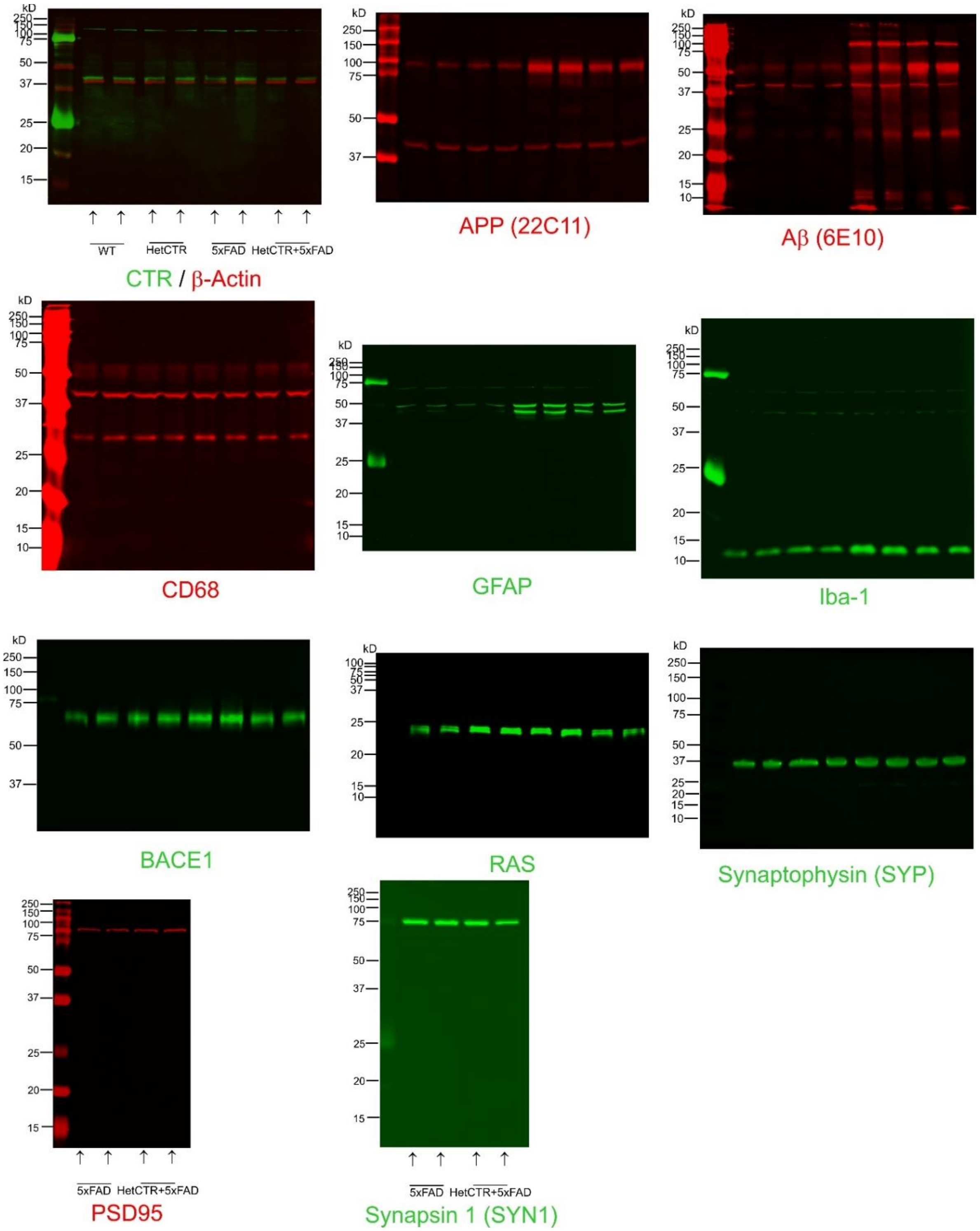

Suppl Fig. 3. Full images of Western blots (using the Li-Cor system) shown in Figure 4.

SI-Table-1. Consumable products and suppliers used in this study.

REAGENT or RESOURCE	SOURCE	IDENTIFIER
**Antibodies**		
β-actin	Sigma	Cat. No. A1978
β amyloid (6E10)	BioLegend	Cat. No. 803001
APP (22C11)	Millipore	Cat. No. MAB348
BACE1	abcam	Cat. No. ab263901
CD31	abcam	Cat. No. ab24590
CD68	abcam	Cat. No. ab201340
CTR	Invitrogen	Cat. No. PA5-25594
GFAP	Life Technologies	Cat. No. 180063
Iba-1	Wako	Cat. No. 016-20001
PSD95 (7E3)	Cell Signaling	Cat. No. 36233
RAMP3	abcam	Cat. No. ab56684, ab197372
RAS	abcam	Cat. No. ab52939
Synapsin 1	abcam	Cat. No. ab64581
Synaptophysin	abcam	Cat. No. ab32127
IRDye 800CW goat anti-rabbit	Li-COR	Cat. No. 926-32211
IRDye 680CW goat anti-mouse	Li-COR	Cat. No. 926-6870
Goat anti-mouse, Alexa Fluor 488	Invitrogen	Cat. No. A-21131
Goat anti-rabbit, Alexa Fluor 594	Invitrogen	Cat. No. A-11012
**Peptides and Chemicals**		
Human Amylin (hAmylin)	AnaSpec, Inc.	Cat. No. AS-50254-1
$A\beta_{1-42}$	rPeptide	Cat. No. A-1002-2
$A\beta_{42-1}$	AnaSpec, Inc.	Cat. No. AS-27275
DAPI (4',6-Diamidino-2-Phenylindole)	Invitrogen	Cat. No. D1306
DMSO	Sigma	Cat. No. D2650-100ml
Evans Blue	Sigma	Cat. No. E-2129
Hexafluoroisopropanol	Sigma	Cat. No. 105228-25G
Polyacrylamide	Bio-Rad	Cat. No. 1610156
SDS	Bio-Rad	Cat. No. 161-0302
Thioflavine S	Sigma	Cat. No. T1892-25G
Triton X-100	VWR	Cat. No. VW3929-2
Odyssey blocking buffer (PBS)	Li-COR	Part No. 927-4000
**Biological Samples**		
HMEC-1	ATCC	Cat. No. CRL-3243
